# The Genome of a Methane-Loving Bacterium

**DOI:** 10.1371/journal.pbio.0020358

**Published:** 2004-09-21

Mention greenhouse gases to most people and they're apt to think of carbon dioxide, fossil fuels, and big cars. Though carbon dioxide emissions are the major source of greenhouse gases, methane is far more effective at trapping heat in the atmosphere. Like increasing carbon dioxide levels, rising levels of atmospheric methane have been attributed to human activity, mostly in the form of landfills, natural gas and oil processing (about 60%), domesticated livestock (cattle account for about 75% of livestock contributions), and rice fields (up to 29% of total emissions).[Fig pbio-0020358-g001]

**Figure pbio-0020358-g001:**
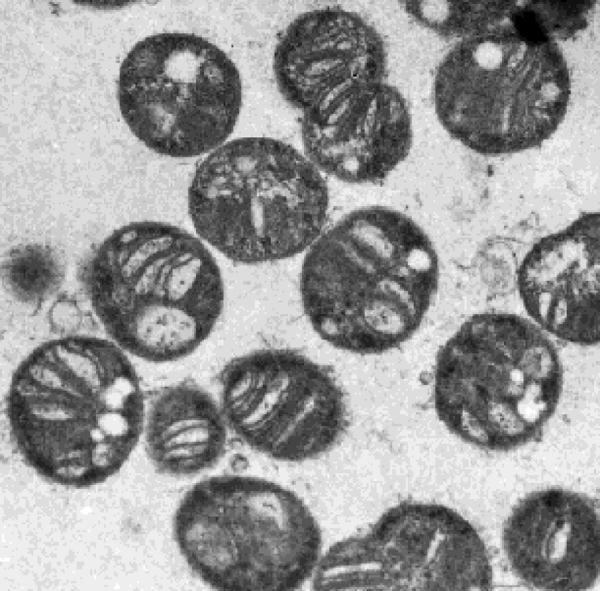
Methylococcus capsulatus cultured in the presence of a high concentration of copper (Image: Anne Fjellbirkeland)

Ruminants—from cows and water buffalo to llamas and vicunas—emit methane gas as a natural by-product of their digestive process, which confers a unique ability to digest cellulose. Ruminants don't digest cellulose directly, but depend on a variety of microbes living in their rumen (main stomach) to do it for them. These microbes ferment cellulose, breaking it down into products the ruminant can digest. During this process, some microbes—bacteria called methanogens—produce methane, which ruminants expel by eructation (otherwise known as belching).

Luckily, there are microbes, called methanotrophs, that consume methane. A type of aerobic bacteria, methanotrophs oxidize methane as an energy and carbon source using the enzyme methane monooxygenase. They've been found in soils, landfills, sediments, hotsprings, and peat bogs, among other environments. Methanotrophs have been the subject of increasing interest because they can use methane as a sole source of carbon and energy—which means they play a major role in global carbon cycles—and could dramatically reduce biologically generated methane emissions. They've also been the focus of bioremediation efforts aimed at environmental decontamination. And now, with the publication of the first complete genome sequence of a methanotroph, such efforts will be all the easier. In this issue of *PLoS Biology,* a multidisciplinary team spanning the fields of genomics, bioinformatics, microbiology, evolutionary biology, and molecular biology report the complete genome sequence of Methylococcus capsulatus and shed light on the metabolism and biology of this ubiquitous microbe.

Contained in a single, circular molecule, the M. capsulatus genome comprises about 3.3 million base pairs—which is about average for a free-living bacterium—with an estimated 3,120 genes. The genome appears well-equipped to meet the specialized needs of this methanotroph, with what appear to be multiple pathways involved in the metabolism of methane and duplications of genes that code for methane monooxygenases, which are essential for the first step of methane oxidation.

Ward et al. also found evidence of “genomic redundancy” in methane oxidation pathways, suggesting that M. capsulatus employs different pathways depending on the availability of molecules needed to sustain cellular activities. Most surprising, the researchers note, was evidence that this methane specialist can turn into a sort of metabolic generalist—with a capacity to use sugars, hydrogen, and sulfur—and appears able to survive reduced oxygen levels. These genome-based hypotheses will require experimental validation, the authors note, but could have important implications for M. capsulatus ecology—including what environments might be amenable to methanotroph-mediated bioremediation.

The genomes of important microbial players in the carbon cycle—including microbes involved in photosynthesis and methanogenesis (methane production)—have already been sequenced. With the addition of a sequenced methanotroph genome, scientists can systematically investigate different genes and regulatory elements to better understand how these methane consumers fit into this global cycle. The M. capsulatus genome provides a platform for investigating the details of methanotroph biology and its potential as a biotech workhorse. It may also guide efforts to harness this bacterium's penchant for methane to reduce global greenhouse gas emissions, to degrade chlorinated hydrocarbons and other persistent pollutants, and to produce protein for animal feed.

